# Versican regulates metastasis of epithelial ovarian carcinoma cells and spheroids

**DOI:** 10.1186/1757-2215-7-70

**Published:** 2014-06-26

**Authors:** Mark Desjardins, Jia Xie, Hilal Gurler, Goda G Muralidhar, Joelle D Sacks, Joanna E Burdette, Maria V Barbolina

**Affiliations:** 1 Departments of Biopharmaceutical Sciences, University of Illinois at Chicago, 833 South Wood Street, PHARM 335, Chicago, IL 60612, USA; 2 Medicinal Chemistry and Pharmacognocy, University of Illinois at Chicago, Chicago, IL 60612, USA

**Keywords:** Ovarian carcinoma, Metastasis, Versican, Adhesion, Migration

## Abstract

**Background:**

Epithelial ovarian carcinoma is a deadly disease characterized by overt peritoneal metastasis. Individual cells and multicellular aggregates, or spheroids, seed these metastases, both commonly found in ascites. Mechanisms that foster spheroid attachment to the peritoneal tissues preceding formation of secondary lesions are largely unknown.

**Methods:**

Cell culture models of SKOV-3, OVCAR3, OVCAR4, Caov-3, IGROV-1, and A2780 were used. In this report the role of versican was examined in adhesion of EOC spheroids and cells to peritoneal mesothelial cell monolayers *in vitro* as well as in formation of peritoneal tumors using an *in vivo* xenograft mouse model.

**Results:**

The data demonstrate that versican is instrumental in facilitating cell and spheroid adhesion to the mesothelial cell monolayers, as its reduction with specific shRNAs led to decreased adhesion. Furthermore, spheroids with reduced expression of versican failed to disaggregate to complete monolayers when seeded atop monolayers of peritoneal mesothelial cells. Failure of spheroids lacking versican to disaggregate as efficiently as controls could be attributed to a reduced cell migration that was observed in the absence of versican expression. Importantly, both spheroids and cells with reduced expression of versican demonstrated significantly impaired ability to generate peritoneal tumors when injected intraperitoneally into athymic nude mice.

**Conclusions:**

Taken together these data suggest that versican regulates the development of peritoneal metastasis originating from cells and spheroids.

## Background

Epithelial ovarian carcinoma (EOC) is a leading cause of death from gynecologic malignancies and the fifth leading cause of death in women [1]. Nearly 90% of all ovarian cancer cases are epithelial in origin and the majority of those belong to a serous histotype. Metastatic disease is highly lethal, and less than 20% of the affected patients survive over a 5 year interval [2,3]. Metastatic progression of EOC is very unique, as metastases that cause death spread locoregionally, in the peritoneal cavity [2]. Malignant cells are shed off of the primary tumor and are carried by the intraperitoneal ascitic fluid, which is followed by implantation at the organs and tissues of the peritoneal cavity, anchorage in submesothelial ECM and establishment of metastases [4,5]. Shed EOC cells may exist as single cells and multicellular aggregates, or spheroids, and both are capable of attaching to the mesothelial layer and transmigrating through outlining peritoneal tissues and organs [6,7].

Successful colonization of the abdomen by EOC cells and spheroids to a large extent depends on their ability to attach to mesothelial surfaces of the peritoneal organs and tissues. EOC cell adhesion to omental “milky spots” composed of various cell types including mesothelial has also been reported [8]. Mesothelial cells are specialized cells that outline the entire surface of the peritoneal cavity. These cells, among other functions, provide a protective barrier against invading pathogens and secrete surfactant molecules to provide a non-adhesive surface. Multiple interactions between EOC cells and mesothelial cells have been reported to contribute to peritoneal adhesion, including CD44-hyaluronan, α5β1-integrin-fibronectin, L1-neuropilin-1, CA125-mesothelin, and CX_3_CL1-CX_3_CR1 [9-16]. Information regarding the mechanisms of EOC spheroid adhesion to the mesothelium is scarce and limited to a single report suggesting the role of β1-integrins in this process [4]. Inhibition of spheroid adhesion could be essential in preventing secondary lesions, as these multicellular aggregates can efficiently escape chemotherapy and radiation, as shown using *in vitro* models, which might contribute to recurrence of EOC in treated patients [4,17-19].

Versican (VCAN) is a secreted proteoglycan protein with multiple functions that can promote tumor metastasis [20,21]. Versican can be expressed in at least 5 different splice variants that were reported to affect cell-cell and cell-matrix adhesion [22-24], migration [25], proliferation, apoptosis [26], and a mesenchymal-epithelial transition [27]. Versican contains several domains [28] that define its binding partners: hyaluronan, integrin, CD44, selectins, EGFR, chemokines, and many others (reviewed in [21]). The exact composition of versican domains varies in each isoform, however the N-terminal hyaluronan-binding and the C-terminal domains are present in all isoforms. Spatial and temporal regulation of versican expression is regulated by very diverse pathways, such as the canonical Wnt/β-catenin signaling [29,30], androgen receptor signaling [31], transcription factor AP-1 [30], microRNA miR-143 [32], and others (reviewed in [33]). Importantly, 50% of tested primary EOC (n = 299) expressed versican [34]. Moreover, overexpression of versican in malignant ovarian stroma is associated with increased invasive potential [35]. Versican could stabilize pericellular matrix and enable stronger adhesion of EOC cells to the mesothelial cells via a CD44-dependent mechanism [15,36]. Furthermore, our previous data demonstrated upregulation of versican in spheroids [37], prompting further studies into the role of this ECM-associated protein in the biology of EOC spheroids and EOC. In this report, we have investigated the role of versican in individual cell and spheroid adhesion, migration and disaggregation *in vitro*, and peritoneal carcinomatosis *in vivo*.

## Methods

### Materials

Matrigel and rat tail collagen type I was obtained from BD Biosciences (Bedford, MA). Human versican shRNA constructs in retroviral GFP vector were obtained from Origene (Rockville, MD). Human versican (pool of 3 proprietary 19 – 25 nt sequences) and control siRNAs were obtained from Santa Cruz Biotechnology (Santa Cruz, CA). DharmaFECT1 was obtained from Dharmacon (Lafayette, CO). Mouse anti-human versican antibody clone 12C5 and mouse anti-human-β-tubulin were obtained from Iowa Developmental Studies Hybridoma Bank (Iowa City, Iowa).

### Cell lines

Human ovarian carcinoma cell lines of serous histotype originating from malignant cells in ascites, OVCAR4, SKOV-3 and A2780, were obtained from the NCI Tumor Cell Repository (Detrick, MD). These cell lines were cultured as suggested by the manufacturer for no longer than twenty consecutive passages. The human ovarian carcinoma cell line of serous histotype originating from malignant cells in ascites, Caov-3, was obtained from Dr. M.S. Stack (University of Notre Dame, ID) and propagated in minimal essential media supplemented with 10% fetal bovine serum (FBS) for no longer than fifteen consecutive passages. OVCAR3 was obtained from ATCC and cultured as recommended. The human ovarian carcinoma cell line of serous histotype originating from a primary tumor, IGROV-1, was obtained from the NCI Tumor Cell Repository (Detrick, MD) and cultured as suggested by the manufacturer for no longer then twenty consecutive passages. The human immortalized peritoneal mesothelial cell line LP-3 was obtained from the Coriell Aging Cell Repository (Camden, NJ) and cultured as indicated by the manufacturer for 5–8 passages. All cell lines were routinely assessed for cellular morphology and average doubling time. All cell lines were propagated from stocks originally obtained from cell banks and individual investigators and have been stored in aliquots for future use. Each aliquot was further propagated for no longer than 20 consecutive passages or 4 months, whichever came first.

### Mice

Athymic nude – FOXN1NU mice were obtained from Harlan Laboratories (Madison, WI) and from Charles River Laboratories (Chicago, IL). All experimental procedures were performed according to the Institutional Animal Care and Use Committee protocol (#10-060) approved by the Animal Care Committee of UIC. Animals were fed ad libitum and maintained in Association for Assessment and Accreditation of Laboratory Animal Care International approved facilities on a 12 h light 12 h dark cycle.

### Transient transfections

EOC cells were cultured to 80% confluence and transfected with siRNAs using DharmaFECT1 according to the manufacturer’s instructions.

### Creating stable clones with reduced VCAN expression

SKOV-3 were transfected with VCAN shRNAs following manufacturer’s suggestions. To create VCAN shRNA-silenced sub cell lines we have used the following four different shRNA sequences designed against multiple splice variants at VCAN gene locus: TGTGACTATGGCTGGCACAAATTCCAAGG, GGATACAGCGGAGACCAGTGTGAACTTGA, GGAAATATCAAGATTGGTCAGGACTACAA, TGGTCATCCAATAGATTCAGAATCTAAAG (Origene Technologies). Several clones have been selected based on their resistance to puromycin and expression of GFP. Residual expression of 5 known isoforms of VCAN has been determined using qPCR resulting in selection of several clones for further experiments.

### Quantitative real-time PCR

Real-time PCR was performed using MyiQ (Bio-Rad) according to the manufacturer’s instructions. Primers for detection of V0, V1, V2, and V3 isoforms of VCAN and a housekeeping gene control eukaryotic translation elongation factor 1 alpha 1 (EEF1A1) have been synthesized using previously reported sequences [38]. The primers for mRNA detection of V4 isoform of VCAN were constructed according to requirements for oligonucleotide primers for quantitative real-time PCR using the Primer3 software. Primer specificity was determined using serial dilutions of the template and by examination of the product melting curves. SYBR Green was used for quantitative PCR as a double-stranded DNA-specific fluorophore. PCR was conducted by initial denaturation for 10 min at 95°C followed by 40 cycles of 94°C for 15 sec and 60°C for 30 sec using the iTaq SYBR Green Supermix (BIO-RAD). To determine the specificity of the PCR primers, the melting curves were collected by denaturing the products at 95°C, then cooling to 65°C, and then slowly melting at 0.5°/sec up to 95°C.

### Spheroid formation

Spheroids were generated using an agarose overlay method described previously [39]. Briefly, non-adhesive agarose plates were prepared by solidifying agarose solution (0.5% in complete culture media) in cell culture plates. EOC cells were released from the monolayers with 0.05% trypsin/EDTA solution, suspended in media containing 2% fetal bovine serum at the concentration of 125,000 cells/ml. 2 ml of this solution was added atop of the solidified agarose and incubated for 48 h at 37°C and 5% CO_2_. Spheroids formed in the suspension and were visualized using bright field microscopy and their diameters measured using AxioVision software (Zeiss). Spheroids were collected from the media with gentle centrifugation for 1 min at 30 × g.

### Cell-cell adhesion and spheroid disaggregation

A human-derived peritoneal mesothelial cell-line LP-3 was cultured in 96-well plates to near confluence. SKOV-3 cells were cultured in monolayers and labeled with fluorescent DiI (Invitrogen) according to the manufacturer’s instructions prior to the adhesion assays. SKOV-3 were subsequently released from monolayers with 0.05% trypsin/EDTA solution and resuspended in serum-free cell culture media. To study adhesion and disaggregation of spheroids, SKOV-3 in monolayers were labeled with DiI and subjected to the spheroid formation assay followed by plating atop of the confluent monolayer of LP-3 (in triplicate per condition) in serum-free media. When needed, SKOV-3 cells were transiently transfected with either control or VCAN-specific siRNAs and used in adhesion assays between 48 and 72 h from the start of transfection. Several clones of SKOV-3 stably transfected with VCAN shRNAs were also used along with the controls stably transfected with scrambled shRNA. Subsequently, the monolayers were washed two times with PBS and fixed in a methanol-containing cell fixative. Adherent cells showing round cell morphology were visualized by red fluorescent signals using a Zeiss fluorescent microscope. The adherent DiI-labeled cells were counted, averaged, and characterized as a percentage from the total. To analyze the role of VCAN in spheroid disaggregation, adherent spheroids were allowed to disaggregate for 24 h followed by outlining the outer perimeter and quantifying the total area taken by the disaggregated spheroid using AxioVision software (Zeiss). Area fold change was calculated by dividing the total area of disaggregated spheroid by that of the spheroid at time zero.

### Cell-ECM adhesion and spheroid disaggregation

Tissue culture-treated 48-well plates were pre-coated with 10 μg/ml human collagen type I and Matrigel (diluted 1:100), or PBS (designated “culture plate”) for 1 h at 37°C. The plates were subsequently rinsed with PBS and air dried. Next, 10 000 SKOV-3 cells were seeded (in triplicate for each condition) in serum-free media in coated wells and allowed to adhere for 5 h at 37°C and 5% CO_2_. This seeding was followed by two washes with PBS, fixation in a methanol-containing cell fixative, and staining. Cells were counted, averaged, and plotted. Spheroids were seeded in serum-free media and allowed to disaggregate for 24 h followed by data analysis including calculation of the area fold change as described above for spheroid disaggregation on LP-3.

### Wound healing assay

To study cell migration using wound healing assays, cells were cultured to complete monolayers in complete media containing 10% FBS followed by 24 h incubation in a media containing no FBS or other growth factors. Wounds were introduced with a plastic pipette tip. Cells cultured in serum-free media were monitored for up to 10 h and photographed using Zeiss AxioVision software. Wound healing was calculated based on the widths of the initial wounds (0 time point) and those at 5 and 10 h and derived as a percentage from the initial. Measurements of the gaps were taken in 10 random places along the wounds, averaged, and percentage of wound healing was calculated based on the lengths of gaps at the initial wounding and at 5 and 10 h, respectively.

### Transwell cell migration

Inserts with 0.8-micron porous membranes were bottom-coated with 1:100 diluted Matrigel for 1 h at 37°C, rinsed, and air-dried. SKOV-3 and IGROV-1 cells (5,000/transwell) in a final volume of 300 μl were seeded in the inserts, which were then placed into 24-well plates filled with serum-free minimal essential media. The cells were allowed to migrate for 5 h at 37°C and 5% CO_2_. Migration was stopped by removing the non-migrated cells from the inside of the inserts. Cells that had migrated through the membranes were fixed, stained, and counted.

### Flow cytometry

The monolayer cells (1×10^6^ per tube) were harvested with trypsin/EDTA and spheroids were harvested with centrifugation and brought to the individual cell state with trypsin/EDTA. Further cells were resuspended in 100 μl of ice cold PBS supplemented with 10% fetal calf serum and 1% sodium azide, fixed in a 1% paraformaldehyde solution in PBS for 15 min on ice, and permeabilized using methanol for 1 h at −20°C. 2 mg of mouse anti-human versican antibody (clone 12C5, Iowa Developmental Studies Hybridoma Bank) was added to the cells. For negative controls, the cells were incubated with either 2 μg of anti-mouse IgG antibody or no primary antibody. Cells were incubated for 1 h on ice in the dark with agitation following washing and resuspension in 400 μl ice cold PBS. 2 μg of goat anti-mouse FITC-conjugated IgG (Millipore) was added to the cells and incubated for 1 h on ice in the dark with agitation. The cells were washed and resuspended in 400 μl of ice cold PBS supplemented with 2% BSA and 1% sodium azide. Labeled cells were analyzed using an Accuri C6 flow cytometer on the same day.

### Immunofluorescence staining

The cells were cultured on glass coverslips to nearly full confluence, fixed, and blocked in goat serum. Mouse anti-human-versican (clone 12C5) antibodies were used at a 1:100 dilution and incubated with cells for 1 h at 22°C. Secondary anti-mouse Alexa433- or anti-mouse Alexa594-conjugated antibodies were used at 1:500 and incubated with cells for 1 h at RT in the dark. 4',6-Diamidino-2-phenylindole (DAPI) was added to the secondary antibody solution to a final concentration of 10 μg/ml 10 min prior to the end of the incubation period. The cells were washed, air dried, and mounted on glass slides using ProlongGold (Invitrogen, Carlsbad, CA). Fluorescent imaging was performed using a Zeiss AxioObserverD.1 fluorescence microscope.

### Western blot

Western blotting analysis was used to detect the expression of versican and β-tubulin in SKOV-3 cells and spheroids. This procedure was performed as previously described [40-42]. Antibodies were used at the following dilutions: 1:100 mouse anti-human-versican (clone 12C5) in 3% BSA in a solution of 50 mM tris-buffered saline, pH 7.4, 150 mM NaCl, and 0.05% Tween-20 (TBST) (Sigma; St. Louis, MO) and 1:200 mouse anti-human-β-tubulin in 3% BSA in TBST. Immunoreactive bands were visualized with an anti-(mouse-IgG)-peroxidase (Sigma, St. Louis, MO) (1:1000 in 3% BSA in TBST), and enhanced chemiluminescence was read using Chemidoc (Bio-Rad) and Bio-Rad Chemidoc ImageReader software.

### In vivo tumor formation

For generation of intraperitoneal tumors 3 × 10^6^ cells/mouse of parental SKOV-3 and SKOV-3 stably expressing VCAN shRNA (2 clones) were used to generate spheroids. Spheroids were injected intraperitoneally (i.p.) into athymic nude mice (n = 6) and animals were monitored three times weekly for tumor formation, ascites development, and survival up to 38 days. To generate intraperitoneal tumors from individual cells, 3 × 10^6^ SKOV-3/mouse were i.p. injected into athymic nude mice (n = 6) and animals were monitored three times weekly for tumor formation, ascites development, and survival for up to 10 weeks. At the end of the experiments animals were sacrificed, dissected, ascites were aspirated, and the abdominal region was examined for tumors. Data analysis was performed as “yes” in case when tumor were visible and “no” when no nodules were seen regardless of the size found at a specific abdominal organ or tissue and plotted as a bar graph depicting the number of animals bearing metastasis at the indicated tissues and organs. Tumors were collected and paraffin-preserved as described earlier [43].

### Statistical analysis of the results of in vivo experiments

The data were treated as coded values for the presence or absence of tumor and compared between the control (SKOV-3) and the SKOV-3 versican shRNA clones 5 and 6 (spheroids). A Chi-square analysis was performed and identified that VCANsh clone6 and VCANsh clone5 were significantly different than the control for all sites in the spheroid groups and VCANsh clone6 was significantly different than the control for all sites in the single cell group (Pearson Chi-square = 13.846, *p* = 0.001, with a Fisher's exact correction at 0.002).

## Results

### Expression of versican is upregulated in EOC spheroids

**Figure 1:**
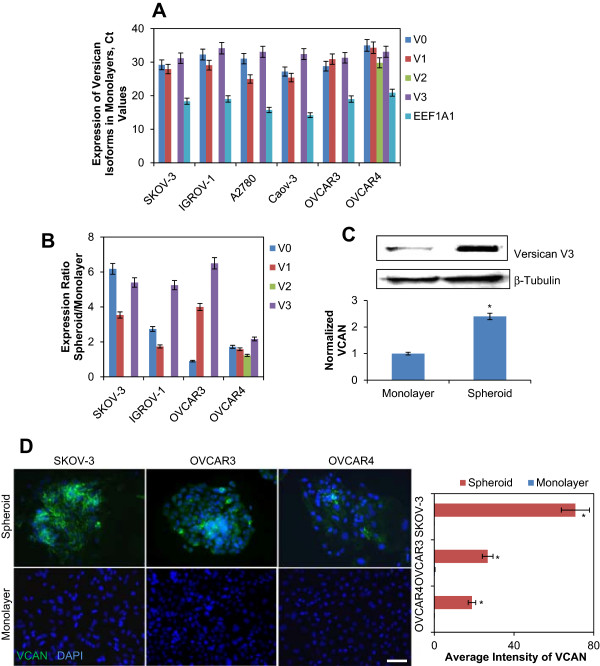
**Expression of VCAN in EOC monolayer cells and spheroids. (A)** Expression of VCAN isoforms V0, V1, and V3 in the indicated EOC cell lines has been tested using QPCR. EEF1A1 served as a housekeeping gene control. **(B)** Expression of VCAN isoforms V0, V1, and V3 was detected in the indicated EOC cell lines cultured in monolayers or as spheroids. Ct values were normalized against those corresponding to the housekeeping gene control EEF1A1. Presented is an average of at least three independent experiments; bars designate the standard deviation. **(C)** Expression of versican V3 in individual cells released from monolayers (termed “single”) and spheroids of SKOV-3 was detected using Western blot. Expression of versican V3 was quantified using optical densitometry, normalized to the expression of a housekeeping gene β-tubulin, and statistically analysed using t-test between spheroid and monolayer groups. **(D)** Expression of versican in monolayers and spheroids of SKOV-3, OVCAR3, and OVCAR4 was assessed using immunofluorescence staining. Bar, 50 micron. Intensity of the versican staining in disaggregated spheroids and monolayers was measured using AxioVert software (Zeiss). Areas occupied by disaggregated spheroids were outlined to measure total intensity of the fluorescence signal, averaged, and plotted in the histogram. Student’s t-test was used to analyze differences in versican fluorescence staining in spheroids and monolayers.

All versican isoforms detected in SKOV-3, IGROV-1, Caov-3, OVCAR3, and A2780 were expressed at different levels (Figure 1A). V2 was expressed only by OVCAR4, while V4 was not detected in any of the EOC cell lines (not shown). V0, V1, and V3 isoforms of versican were upregulated in spheroids of SKOV-3, IGROV-1, and OVCAR3 when compared to the corresponding monolayer cell lines (Figure 1B). These data may suggest the possibility of enhancement of cellular functions facilitated by V0, V1, and V3 in EOC spheroids. Furthermore, a robust versican protein upregulation in spheroids of SKOV-3, OVCAR3, and OVCAR4 was also detected using Western blot and immunofluorescence staining (Figure 1C,D). A recent study compared genomic profiles of ovarian carcinoma and a limited number of human-derived cell lines with the purpose of identifying cell lines with highest genomic similarity to human tumors [44]. Based on those results, OVCAR3 and OVCAR4 were likely derived from high-grade serous ovarian cancer. SKOV3 from ATCC is a model of ovarian cancer, but not high grade, because it is wild-type for p53 [44]. The SKOV3 cell line used in the current study was obtained from the NCI Cell Repository and was different than the cell line studied in [44]. For example, the currently used SKOV3 was null for p53 (Additional file 1: Figure S1A), thus providing further justification for the use of this cell line as a good model as most of EOC contain inactivating mutations in p53 [45]. These cell models were chosen for further examination of versican in EOC spheroids because they displayed increase of versican expression in spheroids compared to monolayer and generated true spheroids that do not come apart upon gentle pipetting. SKOV3 was chosen for functional studies of versican because it replicates well the peritoneal spread of ovarian carcinoma when injected intraperitoneally into abdomens of mice [43].

### Reduction of versican expression affects EOC spheroid and cell adhesion to peritoneal mesothelial cell monolayer and ECM

**Figure 2:**
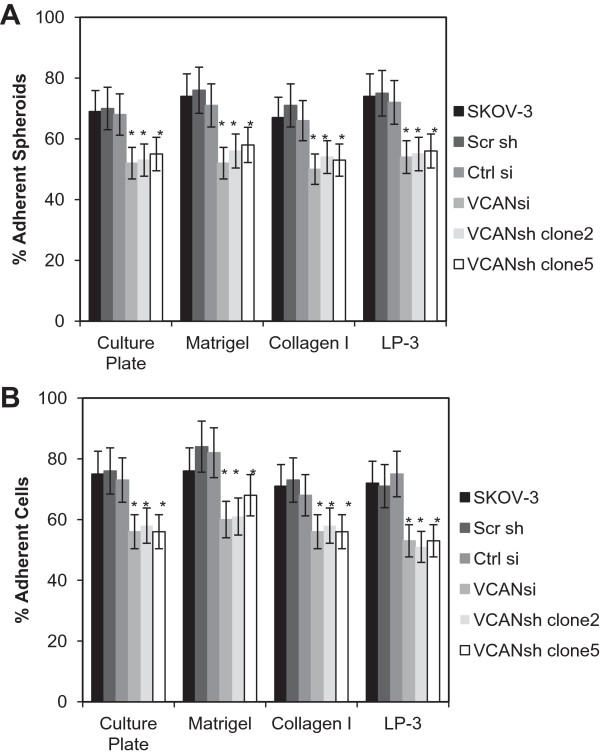
**Versican contributes to adhesion of EOC individual cells and spheroids to the peritoneal mesothelial cells LP-3, Matrigel, and Collagen type I.** Unmodified cell line SKOV-3 and its derivatives stably expressing scrambled shRNA and VCAN shRNA (2 clones were used: clone 2 and clone 5, as indicated), as well as SKOV-3 transiently transfected with either control or VCAN-specific siRNAs were converted into spheroids and seeded **(A)**, or seeded as individual cells **(B)**. Presented is an average of at least three independent experiments; bars designate the standard deviation. *p < 0.05, comparisons were made between experimental (VCANsi, VCANsh) and control (SKOV-3, Scr sh, Ctrl si) groups.

Because versican was upregulated in spheroids (Figure 1A and [37]), its role in EOC spheroid adhesion to mesothelial cell monolayers was monitored. To address this question versican was transiently and stably knocked-down in SKOV-3 or IGROV-1 cells using versican-specific siRNAs and shRNA constructs, respectively. Flow cytometry demonstrated downregulation of versican in VCAN siRNA-transfected cells compared to the controls (Additional file 1: Figure S1B). Residual expression of all isoforms of versican in transiently transfected cells and stably transfected clones was below the detection limit of quantitative RT-PCR (Additional file 1: Figure S1C). Immunofluorescent staining revealed reduced versican expression in VCAN siRNAs transfected cells and clones 5 and 6 generated by stable transfection with shRNAs that was quantitatively analysed using ImageJ software (Additional file 2: Figure S2 and Additional file 3: Figure S3). In order to study adhesion, changes at the terminal time point, 5 h in case of SKOV-3 was chosen, when the majority of cells have adhered, as opposed to studying the kinetics of attachment to different substrates. Both versican-specific siRNA and shRNA were able to reduce adhesion of EOC spheroids to the monolayers of peritoneal mesothelial cells LP-3 by about 25% (Figure 2A). Adhesion of individual EOC cells was also reduced upon siRNA- or shRNA-driven reduction of versican expression (Figure 2B). Furthermore, EOC spheroid and cell adhesion to Matrigel and collagen type I was reduced by about 20-25% as a result of downregulation of versican (Figure 2). These data support a model whereby increased versican expression in spheroids may facilitate effective attachment to peritoneal mesothelial monolayer and underlying ECM.

### Versican regulates spheroid disaggregation on mesothelial cell monolayers

**Figure 3:**
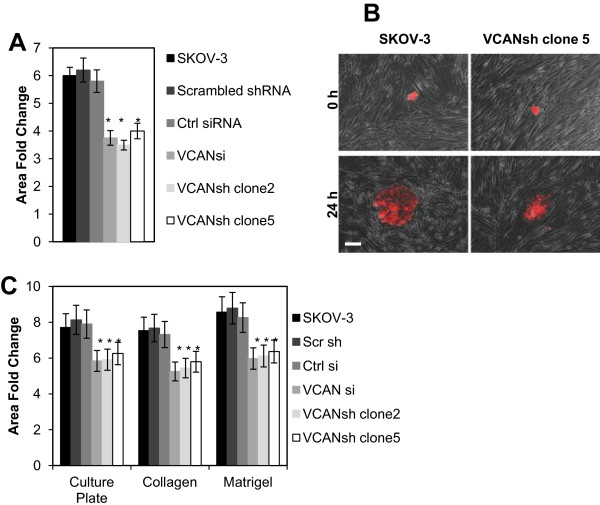
**Versican contributes to EOC spheroid disaggregation. (A)** Unmodified SKOV-3, SKOV-3 stably transfected with scrambled shRNA and VCAN shRNA (2 different clones), and SKOV-3 transiently transfected with control or VCAN siRNA, as indicated, were labeled with fluorescent DiI and used in spheroid formation assay. Measurements of the total area taken by spheroids were taken at 0 and 24 h time points and used for calculation of the area fold change ratios. Presented is an average of at least 3 independent experiments; error bars represent standard deviation; *p < 0.05, comparisons were made between experimental (VCANsi, VCANsh) and control (SKOV-3, Scr sh, Ctrl si) groups. **(B)** A typical image of disaggregating SKOV-3 and SKOV-3 VCAN clone 5 spheroids taken at 0 and 24 h. Bar, 100 micron. **(C)** Unmodified SKOV-3, SKOV-3 stably transfected with scrambled shRNA and VCAN shRNA (2 different clones), and SKOV-3 transiently transfected with control or VCAN siRNA, as indicated, were used in spheroid formation assay. Presented is an average of at least 3 independent experiments; error bars represent standard deviation; *p < 0.05, comparisons were made between experimental (VCANsi, VCANsh) and control (SKOV-3, Scr sh, Ctrl si) groups.

**Figure 4:**
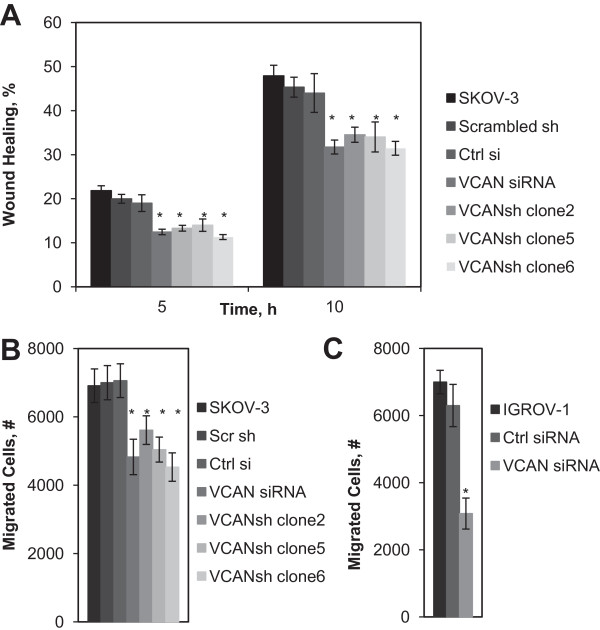
**Versican contributes to EOC cell migration. (A)** Unmodified SKOV-3, SKOV-3 stably transfected with scrambled shRNA and VCAN shRNA (3 different clones), and SKOV-3 transiently transfected with control or VCAN siRNA, as indicated, were cultured to complete monolayers followed wounding with a pipette tip. Presented is an average of at least 3 independent experiments; error bars represent standard deviation; *p < 0.05, comparisons were made between experimental (VCANsi, VCANsh) and control (SKOV-3, Scr sh, Ctrl si) groups. **(B)** Unmodified SKOV-3, SKOV-3 stably transfected with scrambled shRNA and VCAN shRNA (3 different clones), and SKOV-3 transiently transfected with control or VCAN siRNA, as indicated, were seeded in Transwell inserts and allowed to migrate for 5 h. Presented is an average of at least 3 independent experiments; error bars represent standard deviation; *p < 0.05, comparisons were made between experimental (VCANsi, VCANsh) and control (SKOV-3, Scr sh, Ctrl si) groups. **(C)** Unmodified IGROV-1, as well as IGROV-1 transiently transfected with control or VCAN siRNA, as indicated, seeded in Transwell inserts and allowed to migrate for 5 h. Presented is an average of at least 3 independent experiments; error bars represent standard deviation; *p < 0.05, comparisons were made between experimental (VCANsi,) and control (IGROV-1, Ctrl si) groups.

A reduction of versican decreased the ability of EOC spheroids to disaggregate when seeded atop monolayers of peritoneal mesothelial cells LP-3 by approximately 35% (Figure 3A,B). Further, EOC spheroids lacking versican disaggregated on ECM and polystyrene supports yielding significantly smaller total area compared to the controls (Figure 3C). A reduction in total area during disaggregation is an index of the cellular propensity to migrate. Versican isoforms V0, V1, and V3 can contribute to cell migration and metastasis [15,20,24,32,33,46]. In order to address whether versican was required for EOC cell migration, several complimentary methods were used, such as a wound healing assay and Transwell cell migration assay. A reduction of versican slowed the migratory ability of EOC cells (Figure 4). SKOV-3 cells lacking versican migrated about 30% slower compared to the controls (Figure 4A,B), and migration of IGROV-1 in the absence of versican was reduced by approximately 50% (Figure 4C). We investigated whether loss of versican regulates changes in talin expression, as the latter has been demonstrated to play a role in mesothelial monolayer clearance by spheroids [6], and we found that talin RNA and protein remain unchanged (not shown). Altogether these data suggest a possibility that spheroid disaggregation on LP-3 is negatively affected by loss of versican.

### Loss of versican in EOC spheroids reduces formation of peritoneal tumors

**Figure 5:**
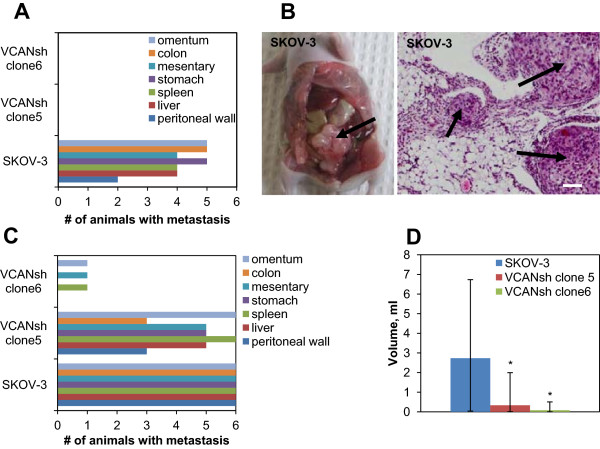
**Versican regulates formation of peritoneal tumors in an *in vivo *xenograft model of EOC. (A)** Parental SKOV-3 and SKOV-3 stably transfected with VCAN shRNA (clone5 and clone6) were used in spheroid formation assay, collected, intraperitoneally injected into abdomens of athymic nude mice at 3 × 10^6^ cells/mouse (n = 6). Animals were monitored 3 times weekly for 38 days following sacrifice, dissection, and documentation of visible tumors. Distribution of metastatic lesions formed on omentum, colon, mesentery, stomach, spleen, liver, and peritoneal wall is shown as the number of animals bearing metastasis at that site. **(B)** Tumors formed after intraperitoneal injection of either transfected with vector control or parental SKOV-3, as indicated. Paraffin-embedded tumors were cut in 5 micron sections and stained with haematoxylin&eosin. Images were generated using Aperio ScanScope; bar, 50 micron. **(C)** Parental SKOV-3 and SKOV-3 stably transfected with VCAN shRNA (clone5 and clone6) were released from monolayers using trypsin, suspended in PBS, and intraperitoneally injected into abdomens of athymic nude mice at 3 × 10^6^ cells/mouse (n = 6). A histograms shows volumes of ascites collected from animals in each experimental and control groups **(D)**. The data were statistically analysed using Student’s t-test; *p < 0.05, comparisons were made between experimental (VCANsh) and control (SKOV-3) groups.

Our *in vitro* data indicated that the loss of versican hindered the ability of EOC cells and spheroids to adhere to peritoneal mesothelial cells, disaggregate, and migrate. All of these affected cellular properties are important for a tumor as well as a metastatic cell. To determine whether the loss of versican observed *in vitro* played a role in any of the steps of formation of peritoneal tumors, an *in vivo* intraperitoneal xenograft model was utilized. Control and versican-deficient spheroids were created using two different clones stably transfected with versican shRNA, respectively. The clones were intraperitoneally injected into the abdomens of athymic nude mice and allowed to seed and develop into peritoneal tumors. Loss of versican in spheroids strongly impacted tumor formation, as both SKOV-3 VCANsh clone5 and SKOV-3 VCANsh clone6 spheroids failed to generate tumors 5.5 weeks following i.p. injection of tumor cells (Figure 5A,B). Passage through the 25 g needle in the course of the intraperitoneal injection did not affect either the integrity of spheroids, or their viability, or their ability to disaggregate (not shown). These data indicate that versican could be one of key players in the process of formation of secondary lesions by EOC spheroids. To further examine the role of versican in peritoneal tumor formation by EOC, we conducted *in vivo* experiments using the same cell lines, i.e. parental SKOV-3, SKOV-3 VCANsh clone5, and SKOV-3 VCANsh clone6, and i.p. injected them into athymic mice abdomens as individual cells in suspension (not spheroids). In this case, the length of the experiment was increased to up to 10 weeks, as this longer endpoint resulted in a phenotype consistent with the terminal point in metastasis formation where animals become moribund and require sacrifice [43]. Although SKOV-3 VCANsh clone5 and SKOV-3 VCANsh clone6 generated intraperitoneal tumors, the overall tumor and ascites formation was robustly reduced (Figure 5C,D). Notably, in SKOV-3 VCANsh clone6 group only one animal generated metastasis at omentum, spleen, and peritoneal wall, and had a small volume of ascites (0.5 ml). In this group the other five animals did not have any visible lesions, did not generate ascites, and were not moribund at 10 weeks post tumor cell injection (Figure 5D). In SKOV-3 VCANsh clone5 group three animals had peritoneal wall and colon metastasis, five had liver, stomach, and mesentery metastasis, and all six had omental and spleen metastasis (Figure 5C). However, in this group the volume of ascites was significantly reduced as compared to the parental SKOV-3 group (Figure 5D). The versican groups overall had a lower tumor burden as all six of the animals in the SKOV3 parental injected group developed tumors at all location sites. Additionally, tumors generated by SKOV-3 VCANsh clone5 were very small (up to 2–10 mm^3^/site) as opposed to tumors in the parental group (>1.5 cm^3^ in total); however, precise measurement of the volumes and sizes was not possible due to overt tumor formation in the parental group. The proliferation rate of SKOV-3 VCANsh clone6 cells was significantly lower than the controls, while SKOV-3 VCANsh clone5 cells proliferated similarly to the controls (Additional file 4: Figure S4). These data may suggest that changes in proliferation could have contributed to formation of tumors from individual cells in SKOV-3 VCANsh clone6 group as compared to the parental SKOV-3 and SKOV-3 VCANsh clone5.

## Discussion

EOC, the deadliest gynecologic cancer, has many unique features that set this malignancy apart from others and make it difficult to treat clinically. The pattern of metastatic spread involves peritoneal organs and occurs via dissemination of the malignant cells from the primary tumor, while lymphagenous and hematogenous metastasis are rare. Peritoneal spread of EOC metastasis is characterized by numerous lesions seeded on various peritoneal tissues and organs, leading to obstruction of bowel, malnutrition, and death. Disseminated EOC cells can exist in the ascites as individual cells and multicellular aggregates, which is another unique feature of this malignancy. Although both are capable of forming metastasis, it has been proposed that spheroids may possess increased invasive ability [47]. Cells forming spheroids are less susceptible to the harmful effects of chemotherapy and radiation *in vitro*, which may allow them to escape treatment and proceed with peritoneal metastases [17-19,39]. Follow-up chemotherapy in EOC becomes significantly less efficient eventually leading to development of incurable metastases. Thus, it is especially important to understand the biology of these potentially more aggressive aggregates in order to prevent or hamper formation of deadly peritoneal metastases. Therapies preventing initial seeding from malignant spheroids could lead to less metastatic lesions, better treatment outcomes, and longer survival. New therapies could be used after surgical resection of primary EOC in early stage patients, as well as for reduction of further spread in patients with advanced disease.

The data presented in this report indicate that a reduction of all forms of versican may abrogate formation of peritoneal lesions seeded by EOC spheroids and it impedes tumor formation by individual cells. Of note, tested cellular properties that ensure success of metastasis, such as peritoneal adhesion, migration, and spheroid disaggregation, were all reduced by only about 30% when versican expression was silenced according to the *in vitro* results. Tumor lesions following intraperitoneal injection of spheroids that were deficient for versican expression demonstrated a delay in seeding such that no lesions were present at five and a half weeks for both clones while tumors could be identified although at a much lower level after ten weeks. These results might lead to a speculation that in some instances it may be sufficient to abrogate multiple metastatic abilities, but not necessarily to completely eliminate them in order to prevent and or delay the formation of metastasis. Alternatively, in the *in vitro* situation, spheroids may precipitate more easily due to gravitational forces, which would allow for their tighter connection with the mesothelial layer. *In vivo*, it is likely that completely different forces may contribute to peritoneal adhesion of spheroids resulting in less successful implantation. Other unknown factors could potentially explain why no lesions after i.p. spheroid injection were formed in our *in vivo* experiments at five and a half weeks, even though they were able to form after 10 weeks and in the *in vitro* studies they were reduced each only by a third. Importantly, spheroids expressing versican (parental SKOV-3 group) were able to form tumors, indicating that the presence of versican is essential for seeding and development of peritoneal lesions. Furthermore, our data suggest that expression of endogenous versican could be important for metastatic progression of EOC along with the stromal versican previously reported by Ghosh [35]. Lastly, in the conditions ensuring that animals injected with parental SKOV-3 become moribund, individual cells lacking versican expression were more successful in forming visible tumors, perhaps, because they had more time to develop visible tumors. These data suggest that versican is a key protein that regulates peritoneal carcinomatosis by cells and spheroids in a xenograft model of EOC.

It remains to be tested in more detail how abrogation of versican in already existent metastasis might result in better outcomes. It is important to mention that versican can promote EOC cell proliferation [35]. However, our data do not fully support that changes in proliferation contribute to formation of tumors in our *in vivo* experiments, as cell proliferation in one of the clones with reduced versican expression was similar to the controls.

At present there are no specific small molecule drugs directed at reduction of versican expression. Nevertheless, other approaches that target the expression and function of versican could be feasible in the future. Our experiments show that versican-specific siRNA and shRNA are effective against formation of EOC peritoneal metastases. These data provide feasibility of this approach provided that future technological discoveries will make it possible to use small RNAs to treat diseases. Another approach that could be attempted in preclinical models is the use of bio-neutralizing antibodies against versican. As versican is a secreted protein associated with the extracellular matrix, use of antibodies, if successful, could target many stages of EOC dissemination starting from peritoneal seeding to the latest stages characterized by expansion of terminal metastasis. While some of these approaches remain to be tested, our data presented here emphasize the potential importance of versican in formation and development of peritoneal metastases of EOC.

## Conclusions

Our data elucidate the expression and role of versican in the formation and development of peritoneal metastases of EOC from both individual cells and spheroids. Our results may also suggest that multiple pro-metastatic cellular functions, such as adhesion and migration, play a significant role in development of metastasis from ovarian carcinoma.

## Abbreviations

EOC: Epithelial ovarian carcinoma; shRNA: Short hairpin ribonucleic acid; VCAN: Versican; EGFR: Epidermal growth factor receptor; ECM: Extracellular matrix; GFP: Green fluorescent protein; PCR: Polymerase chain reaction; EEF1A1: Eukaryotic translation elongation factor 1 alpha1; EDTA: Ethylenediaminetetraacetic acid; FBS: Fetal bovine serum; PBS: Phosphate buffered saline; BSA: Bovine serum albumin; siRNA: Small inhibitory ribonucleic acid.

## Competing interests

The authors declare that they have no competing interests.

## Authors’ contributions

MD, JX, HG, GGM, and JDS contributed to data collection, analysis, and interpretation, JEB contributed to data collection, analysis, drafting and revising of the manuscript. MVB contributed to the conception of the study, data collection, analysis, interpretation, drafting and revising of the manuscript. All authors read and approved the final manuscript.

## Supplementary Material

Additional file 1: Figure S1 Flow cytometry analysis of versican expression in parental non-transfected SKOV-3 as well as those transiently transfected with control (Ctrl) and VCAN siRNAs. (A) Expression of TP53 was tested in EOC cell lines SKOV-3, OVCA432, and OVCAR3 using Western blot. Β-Tubulin was a loading control. (B) Intracellular expression of versican in non-transfected SKOV-3 (Non-transfected), SKOV-3 transfected with control siRNA (Control siRNA), and SKOV-3 transfected with versican-specific siRNAs (VCAN siRNA) was probed with flow cytometry as described in Methods. Black line – cells only, green line – secondary antibody only, red line – isotype control antibody + secondary antibody, blue line – primary anti-versican antibody + secondary antibody. Numbers on the graphs represent percentage of versican-specific species in non-transfected SKOV-3 as well as those transiently transfected with control and VCAN-specific siRNAs. Representative of at least three independent experiments. (C) Expression of VCAN isoforms detected by quantitative PCR in parental SKOV-3 as well as those transiently and stably transfected with either control siRNAs, or VCAN siRNAs, or scrambled shRNAs, or VCAN shRNAs (clones 2, 5, and 6), as indicated. Click here for file

Additional file 2: Figure S2 Immunofluorescence staining of versican in parental non-transfected SKOV-3 as well as those transiently transfected with control (Ctrl) and VCAN siRNAs. (A) Non-transfected SKOV-3 (NT), SKOV-3 transfected with control siRNA (Ctrl si), and SKOV-3 transfected with versican-specific siRNAs (VCAN si) were cultured to a nearly complete monolayer and probed for surface versican expression using anti-versican antibodies (clone 12C5, Iowa Developmental Studies Hybridoma Bank) and anti-mouse Alexa430 (Molecular Probes) as described in Methods. Nuclear DNA was visualized using DAPI. Images were taken using Zeiss AxioObserverD.1 fluorescence microscope using a DAPI and GFP filters for DAPI and versican, respectively, using a built-in black&white camera with a 20 × magnification on the objective. Images were pseudo colored green (for versican) and blue (for DAPI) and superimposed. (B) Intensity of the versican staining was measured using a line scan feature of ImageJ (NIH). Seven vertical lines were drawn in random places across the images, integrated density was determined with the ImageJ software, averaged and plotted on the histogram. Student’s t-test was used to analyze differences in versican staining. Click here for file

Additional file 3: Figure S3 Analysis of extracellular versican levels by immunofluorescence in SKOV-3 stably transfected with scrambled and VCAN siRNAs. (A) SKOV-3 stably transfected with scrambled and versican-specific shRNAs were cultured to a nearly complete monolayer and probed for surface versican expression using anti-versican antibodies (clone 12C5, Iowa Developmental Studies Hybridoma Bank) and anti-mouse Alexa555 (Molecular Probes) as described in Methods. Nuclear DNA was visualized using DAPI. Images were taken using Zeiss AxioObserverD.1 fluorescence microscope using a DAPI and GFP filters for DAPI and versican, respectively, using a built-in black&white camera with a 20 × magnification on the objective. Images were pseudo colored red (for versican) and blue (for DAPI) and superimposed. (B) Intensity of the versican staining was measured using a line scan feature of ImageJ (NIH). Seven vertical lines were drawn in random places across the images, integrated density was determined with the ImageJ software, averaged and plotted on the histogram. Student’s t-test was used to analyze differences in versican staining. Click here for file

Additional file 4: Figure S4 Cell proliferation assay. SKOV-3 stably transfected with vector control, scrambled shRNA, versican shRNA (clones 5 and 6), as indicated, or non-transfected (SKOV-3) were plated at 10% density in 48WP, allowed to attach, starved overnight, and were stimulated with complete media for 24 h followed by WST1 assay as described in Methods. OD430 values were obtained, averaged from at least three independent experiments, plotted, and data were analysed with Student’s t-test. *p < 0.05. Click here for file
