# Supplementary material for: Versican regulates metastasis of epithelial ovarian carcinoma cells and spheroids
Source: J Ovarian Res. 2014 Jun 26;7:70. doi: 10.1186/1757-2215-7-70 (PMC4081460; doi:10.1186/1757-2215-7-70)
Supplement: Additional file 1: Figure S1 — Flow cytometry analysis of versican expression in parental non-transfected SKOV-3 as well as those transiently transfected with control (Ctrl) and VCAN siRNAs. (A) Expression of TP53 was tested in EOC cell lines SKOV-3, OVCA432, and OVCAR3 using Western blot. Β-Tubulin was a loading control. (B) Intracellular expression of versican in non-transfected SKOV-3 (Non-transfected), SKOV-3 transfected with control siRNA (Control siRNA), and SKOV-3 transfected with versican-specific siRNAs (VCAN siRNA) was probed with flow cytometry as described in Methods. Black line – cells only, green line – secondary antibody only, red line – isotype control antibody + secondary antibody, blue line – primary anti-versican antibody + secondary antibody. Numbers on the graphs represent percentage of versican-specific species in non-transfected SKOV-3 as well as those transiently transfected with control and VCAN-specific siRNAs. Representative of at least three independent experiments. (C) Expression of VCAN isoforms detected by quantitative PCR in parental SKOV-3 as well as those transiently and stably transfected with either control siRNAs, or VCAN siRNAs, or scrambled shRNAs, or VCAN shRNAs (clones 2, 5, and 6), as indicated. [file 1757-2215-7-70-S1.pptx]

## Slide 1
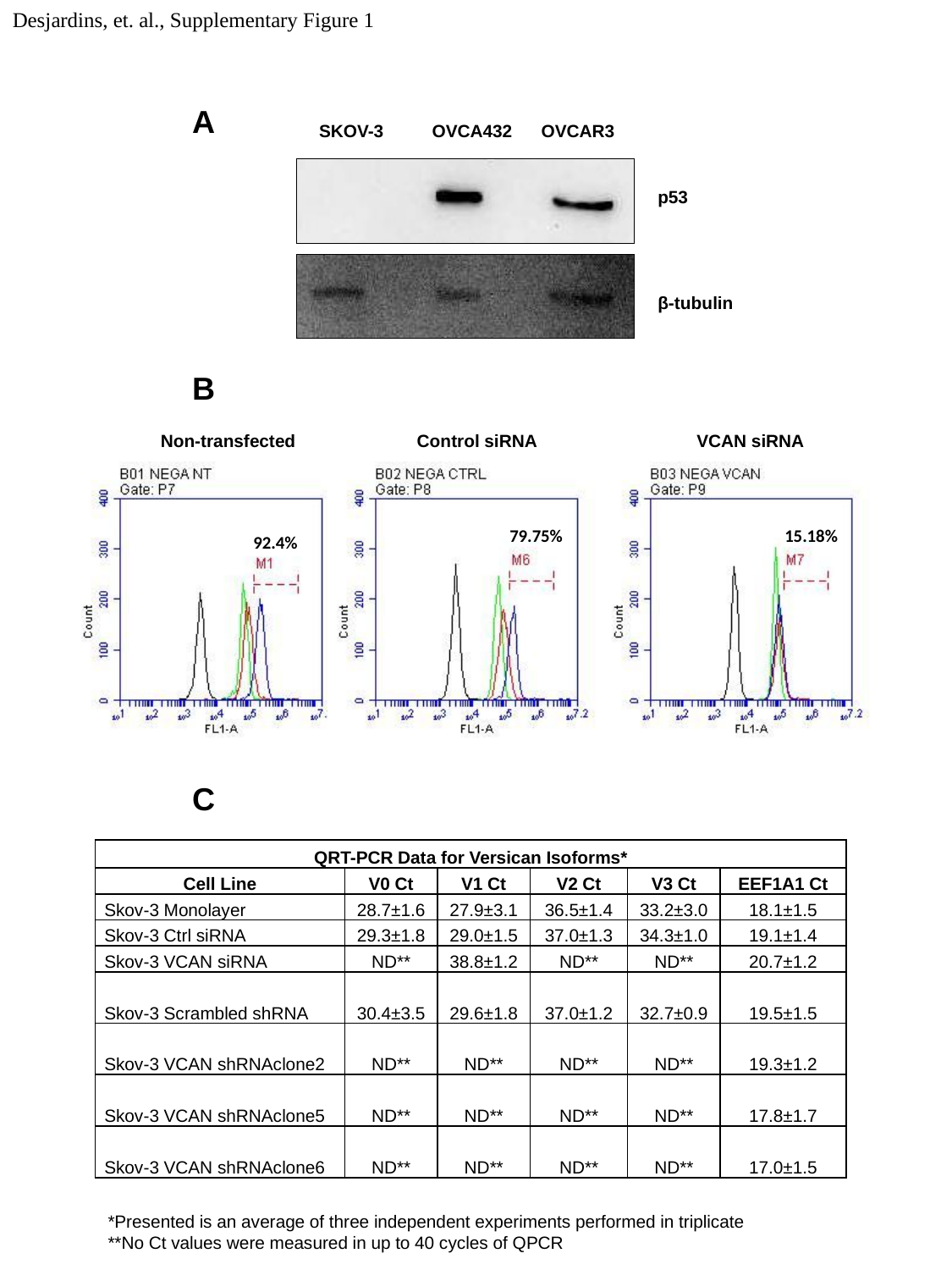

Desjardins, et. al., Supplementary Figure 1
A
SKOV-3 OVCA432 OVCAR3
p53
β-tubulin
B
Non-transfected Control siRNA VCAN siRNA
79.75%
15.18%
92.4%
C
| QRT-PCR Data for Versican Isoforms\* | | | | | |
| --- | --- | --- | --- | --- | --- |
| Cell Line | V0 Ct | V1 Ct | V2 Ct | V3 Ct | EEF1A1 Ct |
| Skov-3 Monolayer | 28.7±1.6 | 27.9±3.1 | 36.5±1.4 | 33.2±3.0 | 18.1±1.5 |
| Skov-3 Ctrl siRNA | 29.3±1.8 | 29.0±1.5 | 37.0±1.3 | 34.3±1.0 | 19.1±1.4 |
| Skov-3 VCAN siRNA | ND\*\* | 38.8±1.2 | ND\*\* | ND\*\* | 20.7±1.2 |
| Skov-3 Scrambled shRNA | 30.4±3.5 | 29.6±1.8 | 37.0±1.2 | 32.7±0.9 | 19.5±1.5 |
| Skov-3 VCAN shRNAclone2 | ND\*\* | ND\*\* | ND\*\* | ND\*\* | 19.3±1.2 |
| Skov-3 VCAN shRNAclone5 | ND\*\* | ND\*\* | ND\*\* | ND\*\* | 17.8±1.7 |
| Skov-3 VCAN shRNAclone6 | ND\*\* | ND\*\* | ND\*\* | ND\*\* | 17.0±1.5 |
*Presented is an average of three independent experiments performed in triplicate
**No Ct values were measured in up to 40 cycles of QPCR
