# Supplementary material for: Versican regulates metastasis of epithelial ovarian carcinoma cells and spheroids
Source: J Ovarian Res. 2014 Jun 26;7:70. doi: 10.1186/1757-2215-7-70 (PMC4081460; doi:10.1186/1757-2215-7-70)
Supplement: Additional file 2: Figure S2 — Immunofluorescence staining of versican in parental non-transfected SKOV-3 as well as those transiently transfected with control (Ctrl) and VCAN siRNAs. (A) Non-transfected SKOV-3 (NT), SKOV-3 transfected with control siRNA (Ctrl si), and SKOV-3 transfected with versican-specific siRNAs (VCAN si) were cultured to a nearly complete monolayer and probed for surface versican expression using anti-versican antibodies (clone 12C5, Iowa Developmental Studies Hybridoma Bank) and anti-mouse Alexa430 (Molecular Probes) as described in Methods. Nuclear DNA was visualized using DAPI. Images were taken using Zeiss AxioObserverD.1 fluorescence microscope using a DAPI and GFP filters for DAPI and versican, respectively, using a built-in black&white camera with a 20 × magnification on the objective. Images were pseudo colored green (for versican) and blue (for DAPI) and superimposed. (B) Intensity of the versican staining was measured using a line scan feature of ImageJ (NIH). Seven vertical lines were drawn in random places across the images, integrated density was determined with the ImageJ software, averaged and plotted on the histogram. Student’s t-test was used to analyze differences in versican staining. [file 1757-2215-7-70-S2.pptx]

## Slide 1
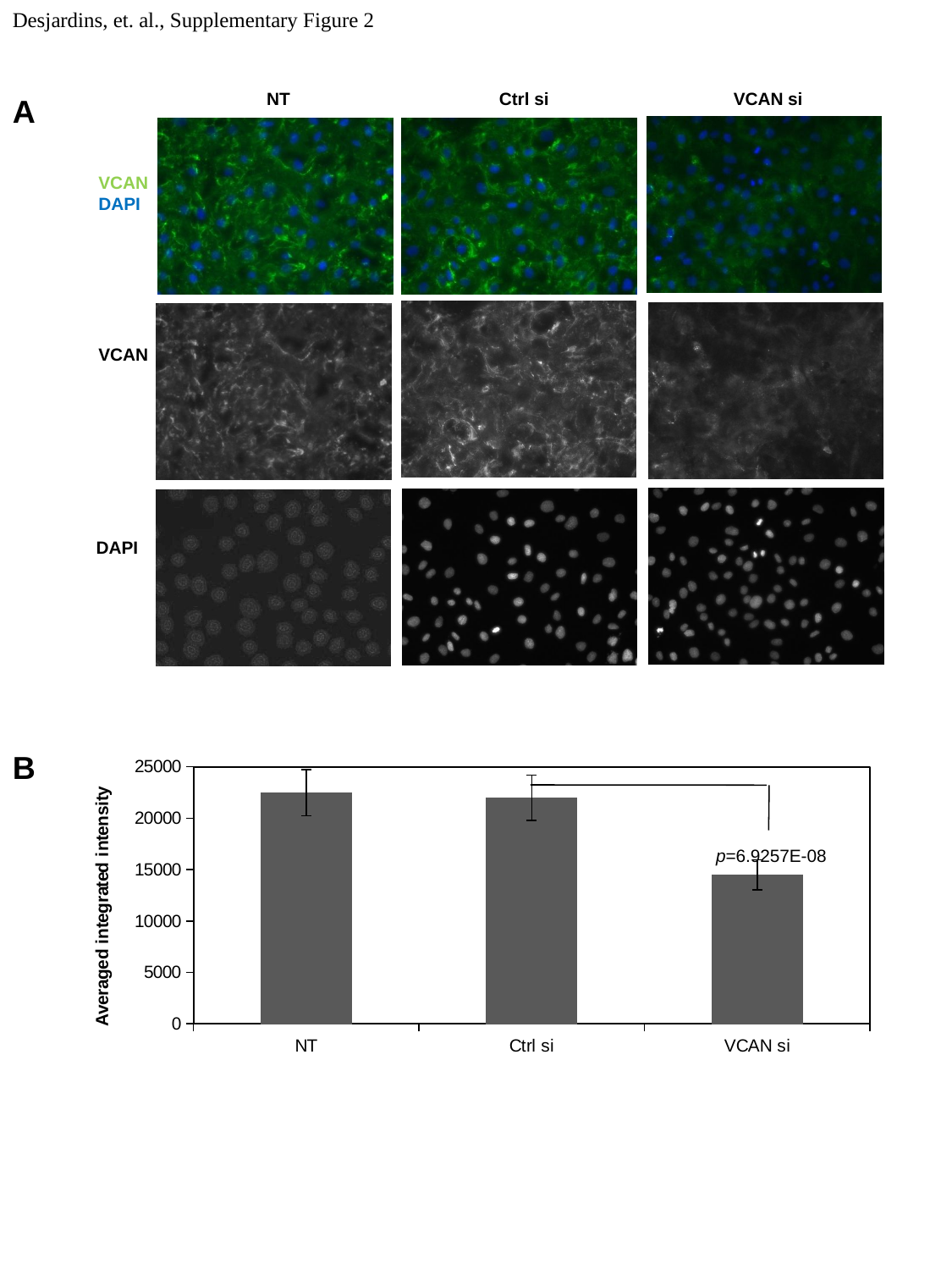

Desjardins, et. al., Supplementary Figure 2
NT Ctrl si VCAN si
A
VCAN
DAPI
VCAN
DAPI
B
### Chart
| Category | Series 1 |
|---|---|
| NT | 22500.0 |
| Ctrl si | 22000.0 |
| VCAN si | 14500.0 |p=6.9257E-08
