# Supplementary figures and images for: Versican regulates metastasis of epithelial ovarian carcinoma cells and spheroids
Source: J Ovarian Res. 2014 Jun 26;7:70. doi: 10.1186/1757-2215-7-70 (PMC4081460; doi:10.1186/1757-2215-7-70)

## Slide 1
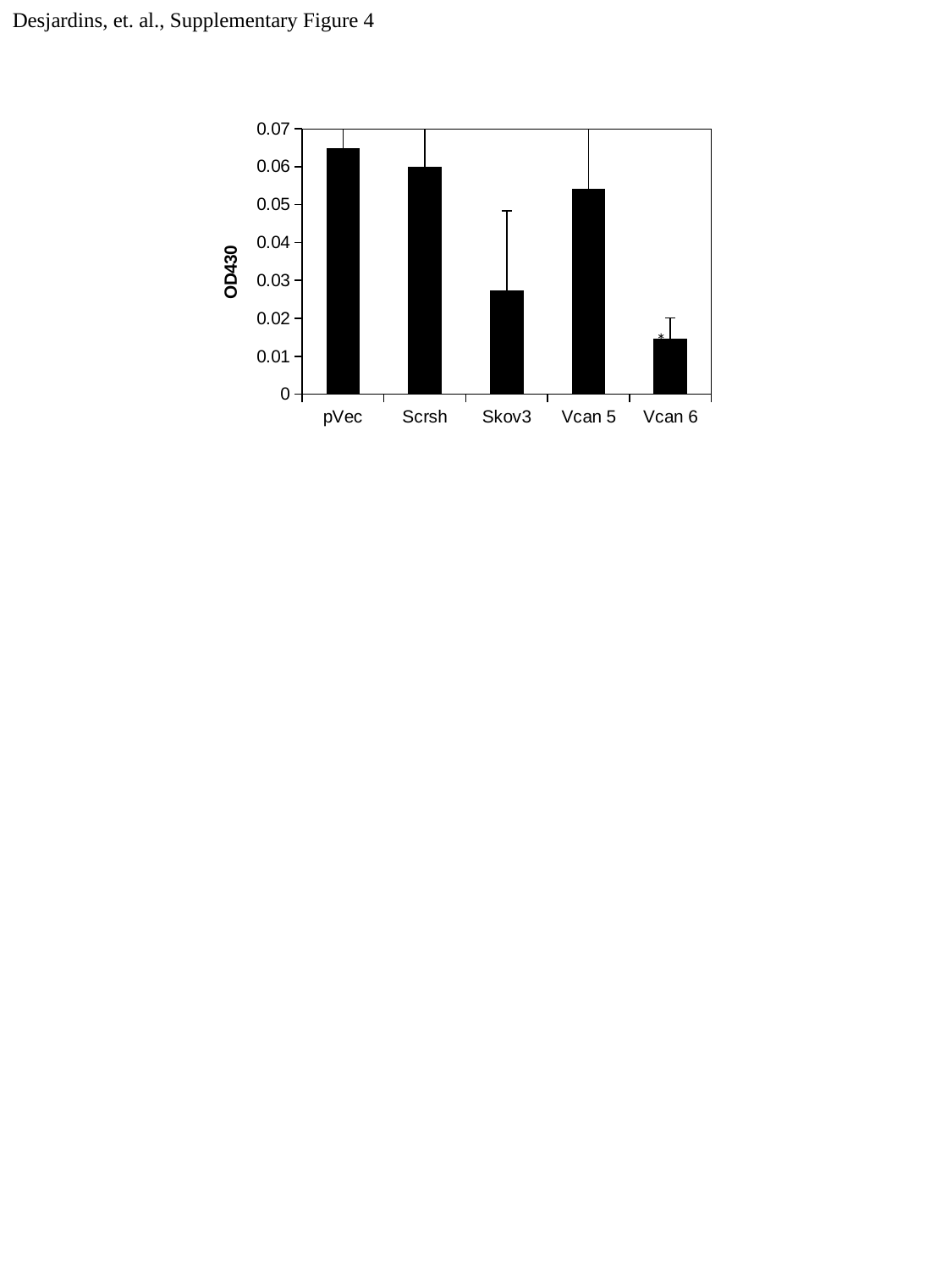

Desjardins, et. al., Supplementary Figure 4
### Chart
| Category | |
|---|---|
| pVec | 0.06480000000000001 |
| Scrsh | 0.05989642857142858 |
| Skov3 | 0.027225000000000003 |
| Vcan 5 | 0.0539875 |
| Vcan 6 | 0.014575 |*

Supplement: Additional file 4: Figure S4 — Cell proliferation assay. SKOV-3 stably transfected with vector control, scrambled shRNA, versican shRNA (clones 5 and 6), as indicated, or non-transfected (SKOV-3) were plated at 10% density in 48WP, allowed to attach, starved overnight, and were stimulated with complete media for 24 h followed by WST1 assay as described in Methods. OD430 values were obtained, averaged from at least three independent experiments, plotted, and data were analysed with Student’s t-test. *p < 0.05. [file 1757-2215-7-70-S4.pptx]
